# Effects of Focal Knee Joint Cooling on Static and Dynamic Strength of the Quadriceps: Innovative Approach to Muscle Conditioning

**DOI:** 10.3390/ijerph18094890

**Published:** 2021-05-04

**Authors:** Joo-Sung Kim, Joni A. Mettler, Kevin McCurdy, Kyung-Min Kim

**Affiliations:** 1 Department of Kinesiology and Sport Sciences, University of Miami, Coral Gables, FL 33146, USA; jjk147@miami.edu; 2 Department of Health and Human Performance, Texas State University, San Marcos, TX 78666, USA; jam388@txstate.edu (J.A.M.); km55@txstate.edu (K.M.); 3 Department of Sport Science, College of Sport Science, Sungkyunkwan University, Suwon-si 16419, Gyeonggi-do, Korea

**Keywords:** isokinetic contraction, cryotherapy, quadriceps strength

## Abstract

Recent evidence suggests an innovative approach to muscle conditioning: focal knee joint cooling (FKJC) appears to improve quadriceps function, including static (isometric) strength. However, there is limited evidence on the effects of FKJC on dynamic (concentric and eccentric) strength. Thus, the purpose of the study was to examine dynamic quadriceps strength following FKJC as well as static strength. Twenty-one college-aged participants volunteered. They randomly underwent 20 min of FKJC and control condition at least 72 h apart. FKJC involves two ice bags, placed on the anterior and posterior surfaces of the knee, whereas the control condition received a plastic ice bag filled with candy corn. We assessed isometric and isokinetic (concentric and eccentric) quadriceps strength at two different velocities (60°/s and 180°/s). Participants performed three maximal voluntary contractions for each mode of muscle contraction, before and after each treatment (immediately, 20, and 40 min after). The outcome variable was maximum knee extension peak torque. FKJC did not change peak torque during any mode of muscle contraction (*p* > 0.05). The current findings suggest that 20 min of FKJC does not change static (isometric) or dynamic (isokinetic) strength of the quadriceps. FKJC was neither beneficial nor harmful to static or dynamic muscular strength.

## 1. Introduction

Quadriceps strength plays a significant role in the proper functioning of the knee joint mechanics as it acts to absorb and distribute intra-articular joint pressures placed at the knee during physical activities [1,2,3]. Quadriceps strength has been shown to contribute to various physical performances [4,5,6,7,8,9]. Specifically, isometric strength has been associated with vertical jump height [5,6], and isokinetic strength showed a positive relationship with hopping [9], vertical jump height [4], and sprinting [7,8]. On the other hand, studies reported that weakness of the quadriceps muscle may result in reduced capabilities of physical activities such as walking, running, and hopping [10,11]. Therefore, improving quadriceps strength is a crucial aspect for physical conditioning.

Cryotherapy, involving the application of a cold agent to the body or muscles, has been frequently used in sports to facilitate faster recovery of the working muscle [12,13,14] or to manage various symptoms such as pain, swelling, and edema after acute injury [15,16,17]. Cryotherapy that decreases tissue temperature is generally believed to have detrimental effects on muscle function because of reduced nerve conduction velocity, muscle spindle sensitivity, or/and muscle strength [18]. These adverse effects were mostly observed when cryotherapy was directly applied to the muscle or whole body, which stiffens muscle fibers and results in a decline in muscle function [19]. However, applying ice bags focally to the knee joint, known as focal knee joint cooling (FKJC), has had a beneficial effect on the quadriceps muscle isometric strength [20,21,22,23], electromyography (EMG) activity during knee extension [20,24], and alpha motor neuron recruitment [25]. For instance, previous studies [20,21,22] have shown that quadriceps maximum voluntary isometric contraction (MVIC) increased by as much as 30% after FKJC. Another study [21] reported that the application of FKJC just before resistance exercise resulted in greater improvement of quadriceps MVIC as compared to performing exercise alone. These promising effects suggest that FKJC should be considered for muscle conditioning.

Improvements in quadriceps function following FKJC may be related to mechanical and/or neuromuscular changes. FKJC that significantly reduces the temperature of the joint, not the muscle tissue, has been found to alter tendon stiffness [18]. For example, 30 min of ice application to the knee joint significantly increased tendon stiffness by more than 25% [26], and tendon stiffness is positively correlated to force output of the knee extensor muscle [27]. In other words, a stiffer tendon facilitates a greater increase in force-production capabilities, suggesting the potential impact of FKJC on quadriceps strength through the tendon–muscle relationship. In addition to this mechanical mechanism, it is assumed that strength increases following FKJC may also be associated with a centrally mediated change in the spinal cord. Hopkins et al. [28] examined the effect of joint cooling on alpha motor neuron activity and muscle strength. They found that 30 min of joint cooling significantly facilitated motor neuron activity and muscle strength, and the improvements lasted for 60 min during the rewarming period [28]. Other studies have also reported an increase in quadriceps motor neuron pool excitability [25], recruitment of motor units [24], and muscle strength [20,21,22] after FKJC application. These reported increases in motor neuron pool excitability, as well as increased tendon stiffness may indicate a potential mechanism for observed increases in quadriceps strength in response to FKJC.

Despite the promising effects of FKJC on quadriceps strength, favorable outcomes [20,21,22] may be limited to isometric actions. A few studies [29,30] investigating the effects of FKJC on isokinetic quadriceps strength failed to observe similar increases in force output that were previously demonstrated during isometric contraction. Rather, they reported reduced strength during isokinetic concentric contraction [29,30]. These contradictory findings may be due to methodological discrepancies between study protocols (subject profile, length of treatment, lack of a control condition, etc.). Therefore, a follow-up experiment is warranted to clarify the effects of FKJC on both the static (isometric) and dynamic (concentric and eccentric) strength of the quadriceps. FKJC may be considered an innovative approach to enhance muscle functions during physical/athletic performance where all modes of muscle contractions are required [7,9,10,28]. If FKJC is found to improve the force output of all muscle contractions, the practical utility would be very high, as ice is very accessible in sport settings, is low-cost, and enables self-conditioning. Therefore, it is important to determine the effects of FKJC on all modes of muscle contraction in a single study. The purpose of the current study was to determine static (isometric) and dynamic (isokinetic) quadriceps strength following FKJC. If we found the expected improvements during isometric contraction that were consistently documented in the literature [20,21,22], the improvement would translate to isokinetic (dynamic) strength because of ice-induced mechanical changes [26] and/or neuromuscular facilitations [25,28]. We hypothesized that FKJC would significantly improve both isometric and isokinetic quadriceps strength as measured by peak torque output.

## 2. Materials and Methods

### 2.1. Design

**Figure 1:**
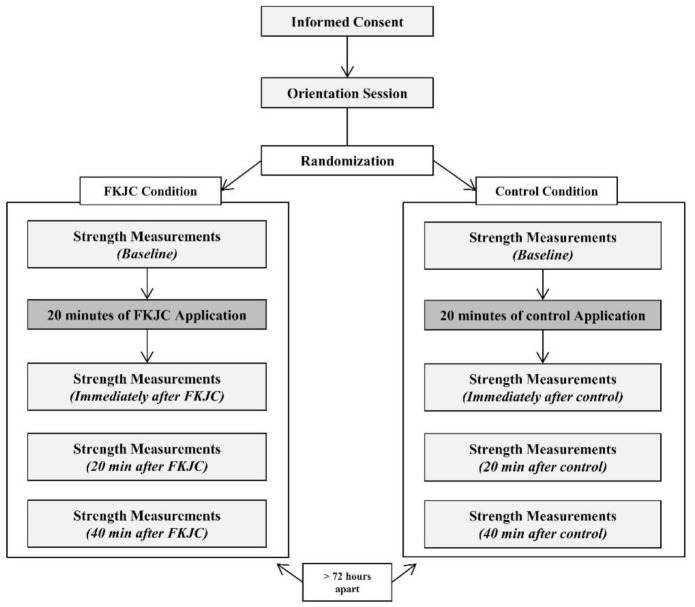
Flow chart of testing procedures.

A randomized crossover design was used to examine the effects of FKJC on quadriceps strength. Quadriceps strength was measured during isometric, concentric, and eccentric contraction at slow (60°/s) and fast (180°/s) velocities. These velocities of contraction during isokinetic testing are associated with functional performance (e.g., hopping, jumping) [31,32]. Quadriceps strength was measured before and after FKJC (immediately, 20, and 40 min after) to examine the change in quadriceps strength over time. Each participant served as their own control. Thus, each participant underwent the FKJC intervention and the control condition with at least a 72-h wash-out period. Skin surface temperature at the involved knee joint and quadriceps muscle was recorded to monitor the delivery of FKJC, and the ambient air temperature was also monitored to maintain the same environmental condition throughout the experiment. Figure 1 illustrates the testing procedure.

### 2.2. Participants

Twenty-one physically active participants (12 men, 9 women; age: 22.7 ± 3.1 years; height: 170.4 ± 10.8 cm; body mass: 74.2 ± 16.4 kg) without current pathology were recruited for this study. To screen for inclusion/exclusion criteria, individuals completed a self-reported questionnaire to assess current and previous history of general medical, neurological, cardiovascular, and orthopedic pathologies. Participants were excluded if they had a history of lower extremity injury within the past six months, neurological disease, impaired circulation, a fear of cryotherapy, allergy to ice, or a history of knee surgery. The sample size was determined using means and standard deviations from a previous study that assessed the effects of FKJC on quadriceps strength during maximal voluntary isometric contraction [22]. Given an alpha of 0.05 and a 1-beta level of 0.80, the effect size of 0.6 was used to calculate the sample size. It was determined that a minimum of 18 participants was required to find statistically significant differences. The G Power software, version 3.1.9.2. was used to calculate the sample size estimate. The study protocol was approved by the Institutional Review Board (ID: 2015F6191) and complied with the Declaration of Helsinki. The study procedures were not performed until informed consent was obtained from each participant.

### 2.3. Procedures

*Familiarization.* The participants were asked to refrain from any resistance exercise from the first visit to the completion of the study in order to produce their best effort during the testing session. The participants were also asked to wear athletic clothes with shorts and not to take any stimulants or medications that may affect the muscle activity within 24 h prior to the testing session. The participants performed isometric, concentric, and eccentric contractions at two contraction velocities (60°/s; 180°/s), which allowed them to become fully accustomed to performing maximal isometric and maximal isokinetic knee extensions. The participants were instructed not to use their trunk and contralateral leg and not to hold the handles to reduce compensatory force-producing strategies during muscle contraction. Additional practice contractions were completed if the participant felt uncertain of how to produce maximal strength. At least 72 h separated the familiarization day and the first testing day.

*Strength Testing.* On each testing day, participants received a brief explanation of the procedure and then were asked to walk at a speed of 2–4 mph on a treadmill for at least 5–10 min as a warm-up. After the warm-up, the participants were seated on an isokinetic dynamometer (Biodex System 4; Biodex Medical Systems, Inc; Shirley, NY, USA) with knee flexion at 80° and hip flexion at 85°. The participants were secured on the dynamometer with straps around the ankle (superior to the malleoli), thigh (midline of the thigh), waist (superior to the anterior superior iliac spine), and shoulders (diagonal from the lateral surface of the clavicle) as instructed by the manufacturer to limit extraneous movements. Then, they proceeded to the practice trials, which consisted of two trials for each type of quadriceps contraction at maximal effort. Participants were then given a 15-min rest period. Maximal strength testing was performed before and after the intervention (immediately, 20 min, and 40 min after intervention application). In the testing session, three maximal isometric contractions were completed with 5 s rest between trials. Participants remained seated on the dynamometer after isometric testing, but the testing posture was adjusted so that knee flexion was at 100° and the hip flexion at 85° for the dynamic concentric and eccentric contractions. During the isokinetic testing, three trials of concentric/eccentric were performed at slow (60°/s) and at a fast (180°/s) velocities in random order with 5 s rest between each trial and two minutes between contraction velocities. A concentric contraction that was immediately succeeded by an eccentric contraction constituted one repetition. The highest peak torque value from the three successful trials for each mode of quadriceps strength (isometric, concentric 60°/s, concentric 180°/s, eccentric 60°/s, and eccentric 180°/s) was used for statistical analysis.

**Figure 2:**
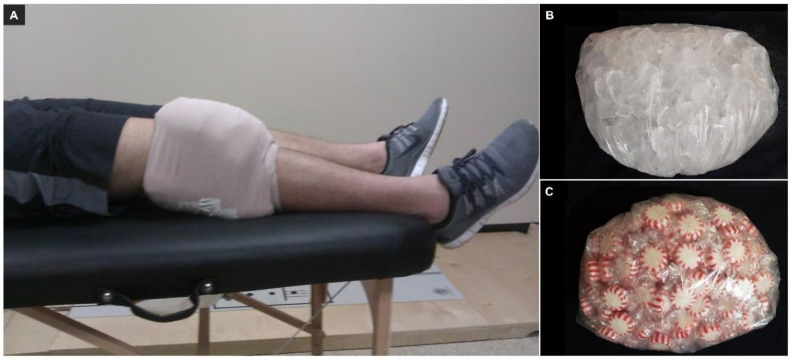
Intervention (**A**) Focal knee joint cooling, (**B**) Ice bag, (**C**) Candy corn bag.

*Intervention.* The participants received either FKJC or control treatment immediately after completion of the baseline quadriceps strength testing, and the intervention order was randomly assigned. The participants were positioned supine on a table to receive the intervention. For FKJC, two 1.5 L plastic bags filled with crushed ice were used, one bag was placed over the anterior surface of the knee (patella), and the other bag was placed over the posterior surface of the knee (popliteal fossa) (Figure 2A,B). Bags were secured on the knee during the intervention using an elastic wrap [22,24]. The control intervention mimicked the FKJC, which used the same type of bag, but the bag was filled with candy corn to control for potential compression effects on strength outcomes (Figure 2C). Both conditions were applied for 20 min. After the first test day, participants were given at least 72 h as a wash-out period. Then, participants returned to the lab to complete the other intervention condition.

**Figure 3:**
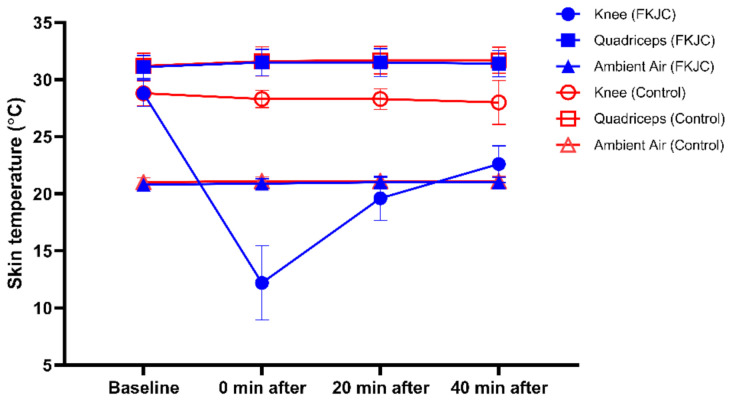
Skin temperature of the knee and quadriceps over a 40-min period following FKJC (blue) and control (red) conditions. FKJC, focal knee joint cooling.

*Skin Temperature Monitoring.* Two surface thermocouples (PT-6; Physitemp Instruments Inc; Clifton, NJ, USA) were used to measure skin surface temperature at two different locations: the center of the patellar tendon and the mid-thigh. Skin temperatures at each location were measured before and after the intervention (immediately, 20, and 40 min after FKJC or control) to monitor the delivery of FKJC to the knee joint, and not the quadriceps [33]. Ambient air temperature in the laboratory was also measured by a surface thermocouple in the same manner as the skin temperatures. The temperature measurements are shown in Figure 3 for descriptive purposes.

### 2.4. Statistical Analysis

Separate two-way repeated-measures analyses of variance (ANOVA) (intervention: FKJC, control treatment; time: before, immediately, 20, and 40 min after intervention) were conducted to compare the effects of FKJC on each of the strength outcomes. Normal distribution was tested by the Kolmogorov–Smirnov test to check the deviation from a normal distribution, and the test confirmed that all dependent variables were normally distributed. Partial eta squared was used to estimate the effect size for strength outcomes. The magnitude of effect size was considered as small (0.01–0.06), moderate (0.06–0.14), and large (>0.14). Alpha level was set a priori at *p* ≤ 0.05. All statistical analyses were conducted with the Statistical Package for Social Sciences (SPSS) software (IBM SPSS v.22, Chicago, IL, USA).

## 3. Results

**Figure 4:**
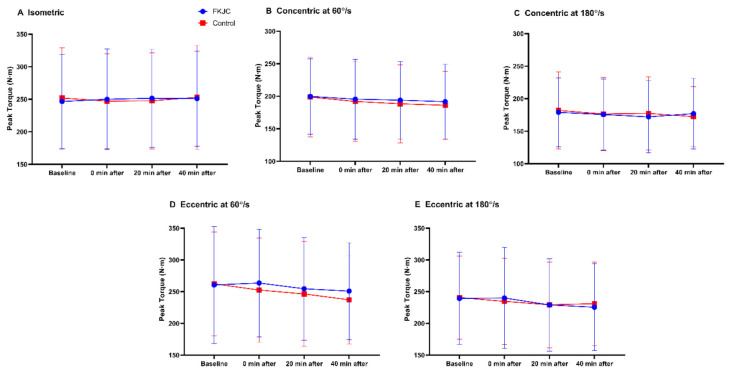
Peak torque data during different modes of quadriceps contractions at baseline, and immediately, 20 min, and 40 min after FKJC (blue) and control (red) condition. FKJC, focal knee joint cooling. (**A**) Isometric, (**B**) Concentric at 60°/s, (**C**) Concentric at 180°/s, (**D**) Eccentric at 60°/s, (**E**) Eccentric at 180°/s. Data are presented as mean ± standard deviation.

**Figure 5:**
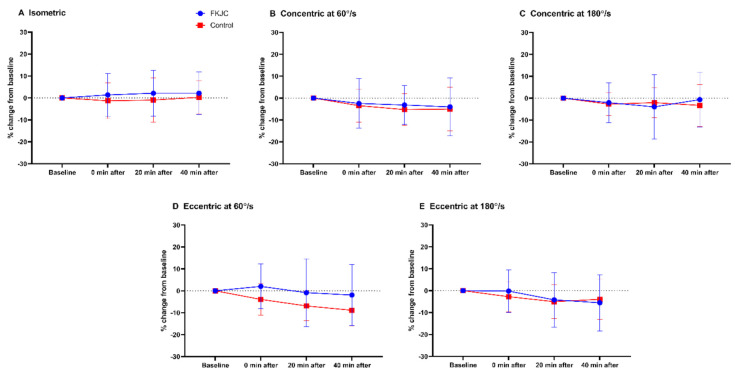
Percent change in peak torque from baseline during different modes of quadriceps contraction after FKJC (blue) and control (red) condition. FKJC, focal knee joint cooling. (**A**) Isometric, (**B**) Concentric at 60°/s, (**C**) Concentric at 180°/s, (**D**) Eccentric at 60°/s, (**E**) Eccentric at 180°/s. Data are presented as mean ± standard deviation.

Figure 4 shows peak torque data of all contraction modes (presented as mean values ± standard deviation) before and after intervention (immediately, 20, and 40 min after the intervention). Figure 5 illustrates percent change in peak torque from baseline during different modes of quadriceps contraction after intervention for the descriptive purpose.

*Isometric Contraction.* We did not observe a significant time x intervention interaction effect (*F*_(3,60)_ = 0.667, *p* = 0.575, (eta)^2^ = 0.032) or intervention main effect (*F*_(1,20)_ = 0.000, *p* = 0.985, (eta)^2^ = 0.000) (Figure 4A).

*Concentric Contraction.* For the contraction velocity at 60°/s, there was no time x intervention interaction effect (*F*_(3,60)_ = 0.448, *p* = 0.720, (eta)^2^ = 0.022) and no significant main effect for intervention (*F*_(1,20)_ = 0.813, *p* = 0.378, (eta)^2^ = 0.039) (Figure 4B). For the contraction velocity at 180°/s, we did not observe a significant time x intervention interaction effect (*F*_(2,42)_ = 1.249, *p* = 0.299, (eta)^2^ = 0.059) or intervention effect (*F*_(1,20)_ = 0.119, *p* = 0.733, (eta)^2^ = 0.006) (Figure 4C).

*Eccentric Contraction.* For the contraction velocity at 60°/s. there was no significant time x intervention interaction effect (*F*_(2,44)_ = 1.810, *p* = 0.172, (eta)^2^ = 0.083) or intervention main effect (*F*_(1,20)_ = 3.033, *p* = 0.097, (eta)^2^ = 0.132) (Figure 4D). For the contraction velocity at 180°/s, there was no significant time x intervention interaction effect (*F*_(3,60)_ = 0.860, *p* = 0.467, (eta)^2^ = 0.041) or intervention main effect (*F*_(1,20)_ = 0.008, *p* = 0.930, (eta)^2^ = 0.000) (Figure 4E).

## 4. Discussion

To the best of our knowledge, this study is the first to examine a comprehensive strength profile of isometric strength and concentric and eccentric quadriceps strength at angular velocities of 60°/s and 180°/s following FKJC. We found that the use of FKJC did not lead to an increase in maximal force output of the knee joint as represented by peak torque during either isometric or isokinetic knee extension contractions.

Our findings regarding isometric strength following FKJC are not in agreement with previous studies [20,21,22]. Pietrosimone et al. [22] found a 10% increase of isometric quadriceps strength after 20 min of FKJC in healthy individuals, and this effect remained until 45 min after FKJC. For instance, baseline peak torque increased from 263 N∙m to 289 N∙m immediately after FKJC and remained elevated until 45 min after the intervention. In contrast, the current study observed only a trivial and non-significant change (3.5 to 4.4 N∙m increase) after FKJC. Pietrosimone et al. [22] suggested that FKJC can induce greater activation of the motor neuron pool, resulting in additional motor unit recruitment, which plays a role in increasing the force production of quadriceps. Different methodological characteristics and low statistical power from the previous study [22] may explain the contradictory results of the effect of FKJC on isometric strength. For instance, in the study by Pietrosimone et al. [22], an isometric quadriceps contraction was performed at 70° of knee flexion. In contrast, the present study utilized 80° of knee flexion. In maximal voluntary isometric contraction, discrepancy in knee joint angles may lead to differences in the torque–joint angle relationship of the quadriceps muscle [34,35]. One of the unique characteristics of isometric contraction is that the contractile elements of the muscle shorten while the elastic elements (tendinous structures) at the ends of the muscle fibers are pulled (increased tension) until the muscle reaches maximal force generation [36]. In this regard, the contractility of the quadriceps muscle during maximal isometric contraction may differ depending on the position of knee flexion angle during testing, which is associated with a change in tendon stiffness. Tendon stiffness appears to be intensified when cooling is applied to the knee joint [26]. Previous studies reported that 30 min of local cooling of the knee joint significantly increased tendon stiffness, and there was a positive relationship between stiffness and quadriceps torque production [26,27]. Marginson et al. [34] showed different isometric quadriceps force outputs depending on the knee flexion angle. For instance, in the same study [34], even a 10° difference in knee flexion angle during the isometric contraction led to a significant change in the force output [34]. Thus, despite the relatively small difference in knee flexion angle (10°) during isometric contractions between the current study and previous study [22], considering the possible impacts of joint cooling on the muscle–tendon relationship, FKJC may differently influence isometric peak torque associated with a slight difference in knee joint angle. The previous study [22] showed that isometric peak torque was significantly improved immediately after 20 min of FKJC application compared with the control condition. However, this statistically significant finding may be influenced by a concurrent decrease in peak torque following the control condition from the baseline measurement. Lastly, the previous study [22] had a low statistical power to detect differences between FKJC and control conditions. Therefore, it is possible that the effects of FKJC on isometric quadriceps strength in healthy individuals is either minimal or absent according to the findings of the current and previous investigations [22].

Different subject populations between studies may also account for different outcomes for isometric quadriceps strength following FKJC. Previous studies [20,21] using a pathological knee model have shown an increase in isometric strength after cooling. For instance, Rice et al. [21] found that isometric quadriceps strength increased by 16% following FKJC in participants with arthrogenic muscle inhibition (i.e., muscle activation failure by neural inhibition) induced by using an experimental model of knee swelling. Additionally, Loro et al. [20] found an increase in isometric quadriceps strength after FKJC in individuals after recent anterior cruciate ligament or meniscus repair surgery. It is suggested that quadriceps strength changes after FKJC can be attributed to the disinhibitory effect of FKJC, which excites cutaneous receptors including mechanoreceptors and thermoreceptors. Such stimulation plays a role in masking inhibitory signaling to the central nervous system [37] by facilitating motor neuron pool of the quadriceps. However, this disinhibitory model is not likely applicable to healthy individuals without joint pathology, as no pain or swelling is present to inhibit the activation of surrounding muscles. Thus, it is possible that the effect of FKJC on isometric quadriceps strength may be limited to individuals with the presence of muscle inhibition caused by joint injury or other joint pathology.

The present study showed that FKJC did not affect concentric quadriceps strength, and our findings contradict some previous work [30] that found a negative impact of FKJC on force output during concentric contraction in healthy individuals. Rhodes et al. [30] reported a significant reduction (10–20%) of concentric quadriceps strength measured by peak torque following FKJC. They suggested that the cooling causes muscle stiffness and may lead to reduced muscle strength by altering the mechanical properties of the tissue and desensitization of the mechanoreceptors. [30] However, whether the cooling application affects the joint receptors is still in question. Furmanek et al. [38] examined the proprioceptive activities of the knee joint as assessed by sense of joint position and force production accuracy (variability in force production) of 66 healthy subjects following FKJC, which was applied over the knee joint and the quadriceps muscle for 20 min. They found no significant reduction of joint position sense and force production accuracy after FKJC, suggesting no adverse effect of FKJC on the sensitivity of these receptors [38]. Joint stability is dependent on muscle strength and proprioception. Sufficient neuromuscular control is required to maintain joint stability, and muscle performance will not be maintained when stability of the joint is impaired. Therefore, given that FKJC did not appear to alter activity of the joint receptors, it may be plausible that concentric peak torque is not negatively affected by the cooling application. However, the sensitivity of joint receptors after FKJC requires further investigation in order to understand the underlying mechanisms of joint stability and the force-generating relationship, especially during concentric contraction following FKJC.

The current study findings are consistent with a previous study [39] with regard to eccentric strength. Kimura et al. [39] found that cryotherapy did not alter the eccentric strength at slow (30°/s) and relatively fast (120°/s) contraction velocities at the ankle joint following application of cold via ice bath immersion (leg immersion in a 10 °C ice bath). Although no study directly examined eccentric quadriceps strength at slow (60°/s) and fast (180°/s) velocities following FKJC, a previous study [40] on athletic performance after FKJC was also in line with the current findings. It was found that a squat jump, which requires the synergetic function of concentric and eccentric quadriceps strength, was not affected by FKJC [40]. Findings of the current study, in addition to previous studies [39,40], demonstrate that FKJC interventions result in little to no eccentric quadriceps performance enhancement.

There are limitations in the current study. The temperatures of knee joint and quadriceps were measured extrinsically. However, skin surface temperature is associated with intra-articular temperature of the knee [41]; therefore, it is assumed that FKJC successfully reduced the temperature of the knee joint. This study is also limited to single-joint exercises as it is possible that the response to multi-joint exercises (i.e., squats and jumps) may be different following FKJC. Lastly, FKJC was limited to the application at the joint. Thus, the intervention may not be generalizable to the muscle performance following other types of cryotherapy.

Our findings of no change in strength following FKJC indicate that the static (isometric) and dynamic (isokinetic) strength of the quadriceps are not negatively affected by FKJC, indicating that the use of FKJC may be important to coaches who are involved in a sport setting where a primary muscle at work is the quadriceps (i.e., cycling, track, and field, etc.). Cryotherapy may be a safe modality to condition muscles due to its physiological benefits (i.e., attenuation of central fatigue [42] and promotion of recovery [43]). Additionally, FKJC could be used in physical rehabilitation before active strengthening exercise (i.e., cryokinetics) as a means to improve muscle strength while also providing analgesic effects [23,44]. A recent meta-analysis [42] revealed that cryotherapy application before exercise appeared to be effective in maintaining muscle performance during prolonged muscle contraction (i.e., running and cycling) [42]. However, it should be noted that cryotherapy may negatively affect maximal muscle strength when applying it directly to the muscles [19]. Therefore, when applying cryotherapy for joint cooling, it is important to localize the cooling to the joint area only so that the surrounding musculature will not be negatively affected.

## 5. Conclusions

We found that 20 min of FKJC did not alter isometric, concentric, and eccentric quadriceps strength at slow and fast contraction velocities. These results suggest that using FKJC was neither beneficial nor harmful to the static and dynamic strength of the quadriceps. The current study provides support for evidence-based practice for sports healthcare practitioners and coaches who consider FKJC as a treatment modality for managing pain and/or acute injuries [17,18] without attenuating quadriceps function.

## Data Availability

The raw data of this study will be available at reasonable request.

## References

[1] Harkey M.S., Gribble P.A., Pietrosimone B.G. (2014). Disinhibitory Interventions and Voluntary Quadriceps Activation: A Systematic Review. J. Athl. Train..

[2] Thompson J.A., Chaudhari A.M.W., Schmitt L.C., Best T.M., Siston R.A. (2013). Gluteus Maximus and Soleus Compensate for Simulated Quadriceps Atrophy and Activation Failure during Walking. J. Biomech..

[3] Yeow C.H. (2013). Hamstrings and Quadriceps Muscle Contributions to Energy Generation and Dissipation at the Knee Joint during Stance, Swing and Flight Phases of Level Running. Knee.

[4] Fischer F., Blank C., Dunnwald T., Gfoller P., Herbst E., Hoser C., Fink C. (2017). Isokinetic Extension Strength Is Associated With Single-Leg Vertical Jump Height. Orthop. J. Sports Med..

[5] Morrissey M.C., Harman E.A., Johnson M.J. (1995). Resistance Training Modes: Specificity and Effectiveness. Med. Sci. Sports Exerc..

[6] De Ruiter C.J., Van Leeuwen D., Heijblom A., Bobbert M.F., de Haan A. (2006). Fast Unilateral Isometric Knee Extension Torque Development and Bilateral Jump Height. Med. Sci. Sports Exerc..

[7] Dowson M.N., Nevill M.E., Lakomy H.K., Nevill A.M., Hazeldine R.J. (1998). Modelling the Relationship between Isokinetic Muscle Strength and Sprint Running Performance. J. Sports Sci..

[8] Cometti G., Maffiuletti N.A., Pousson M., Chatard J.C., Maffulli N. (2001). I Isokinetic Strength and Anaerobic Power of Elite, Subelite and Amateur French Soccer Players. Int. J. Sports Med..

[9] Lee S.E.K., Lira C.A.B., Nouailhetas V.L.A., Vancini R.L., Andrade M.S. (2018). Do Isometric, Isotonic and/or Isokinetic Strength Trainings Produce Different Strength Outcomes?. J. Bodyw. Mov..

[10] Keays S.L., Bullock-Saxton J.E., Newcombe P., Keays A.C. (2003). The Relationship between Knee Strength and Functional Stability before and after Anterior Cruciate Ligament Reconstruction. J. Orthop. Res..

[11] Knoll Z., Kiss R.M., Kocsis L. (2004). Gait adaptation in ACL Deficient Patients before and after Anterior Cruciate Ligament Reconstruction Surgery. J. Electromyogr. Kinesiol..

[12] Hausswirth C., Louis J., Bieuzen F., Pournot H., Fournier J., Filliard J.R., Brisswalter J. (2011). Effects of Whole-Body Cryotherapy vs. Far-Infrared vs. Passive Modalities on Recovery from Exercise-Induced Muscle Damage in Highly-Trained Runners. PLoS ONE.

[13] Fonda B., Sarabon N. (2013). Effects of Whole-Body Cryotherapy on Recovery after Hamstring Damaging Exercise: A Crossover Study. Scand. J. Med. Sci. Sports.

[14] Bailey D.M., Erith S.J., Griffin P.J., Dowson A., Brewer D.S., Gant N., Williams C. (2007). Influence of Cold-Water Immersion on Indices of Muscle Damage following Prolonged Intermittent Shuttle Running. J. Sports Sci..

[15] Banfi G., Lombardi G., Colombini A., Melegati G. (2010). Whole-Body Cryotherapy in Athletes. Sports Med..

[16] Bleakley C.M., McDonough S.M., MacAuley D.C., Bjordal J. (2006). Cryotherapy for Acute Ankle Sprains: A Randomised Controlled Study of Two Different Icing Protocols. Br. J. Sports Med..

[17] Lessard L.A., Scudds R.A., Amendola A., Vaz M.D. (1997). The Efficacy of Cryotherapy Following Arthroscopic Knee Surgery. J. Orthop. Sports Phys. Ther..

[18] Swenson C., Sward L., Karlsson J. (1996). Cryotherapy in Sports Medicine. Scand. J. Med. Sci. Sports.

[19] Bleakley C.M., Costello J.T., Glasgow P.D. (2012). Should Athletes Return to Sport After Applying Ice? A Systematic Review of the Effect of Local Cooling on Functional Performance. Sports Med..

[20] Loro W.A., Thelen M.D., Rosenthal M.D., Stoneman P.D., Ross M.D. (2019). Effects of Cryotherapy on Quadriceps Electromyographic Activity and Isometric Strength in Patient in the Early Phases following Knee Surgery. J. Orthop. Surg..

[21] Rice D., McNair P.J., Dalbeth N. (2009). Effects of Cryotherapy on Arthrogenic Muscle Inhibition Using an Experimental Model of Knee Swelling. Arthritis Rheum..

[22] Pietrosimone B.G., Ingersoll C.D. (2009). Focal Knee Joint Cooling Increases the Quadriceps Central Activation Ratio. J. Sports Sci..

[23] Hart J.M., Kuenze C.M., Diduch D.R., Ingersoll C.D. (2014). Quadriceps Muscle Function after Rehabilitation with Cryotherapy in Patients with Anterior Cruciate Ligament Reconstruction. J. Athl. Train..

[24] Pietrosimone B.G., Hart J.M., Saliba S.A., Hertel J., Ingersoll C.D. (2009). Immediate Effects of Transcutaneous Electrical Nerve Stimulation and Focal Knee Joint Cooling on Quadriceps Activation. Med. Sci. Sports Exerc..

[25] Hopkins J.T., Ingersoll C.D., Edwards J., Klootwyk T.E. (2002). Cryotherapy and Transcutaneous Electric Neuromuscular Stimulation Decrease Arthrogenic Muscle Inhibition of the Vastus Medialis after Knee Joint Effusion. J. Athl. Train..

[26] Alegre L.M., Hasler M., Wenger S., Nachbauer W., Csapo R. (2016). Does Knee Joint Cooling Change in Vivo Patellar Tendon Mechanical Properties?. Eur. J. Appl. Physiol..

[27] Bojsen-Moller J., Magnusson S.P., Rasmussen L.R., Kjaer M., Aagaard P. (2005). Muscle Performance during Maximal Isometric and Dynamic Contractions is Influenced by the Stiffness of the Tendinous Structures. J. Appl. Physiol..

[28] Hopkins J.T., Stencil R. (2002). Ankle Cryotherapy Facilitates Soleus Function. J. Orthop. Sports Phys..

[29] Alexander J., Rhodes D. (2019). Temporal Patterns of Knee-Extensor Isokinetic Torque Strength in Male and Female Athletes Following Comparison of Anterior Thigh and Knee Cooling Over a Rewarming Period. J. Sport Rehabil..

[30] Rhodes D., Alexander J. (2018). The Effect of Knee Joint Cooling on Isokinetic Torque Production of the Knee Extensors: Considerations for Application. Int. J. Sports Phys. Ther..

[31] Negrete R., Brophy J. (2000). The Relationship Between Isokinetic Open and Closed Chain Lower Extremity Strength and Functional Performance. J. Sport Rehabil..

[32] Atabek H.Ç., Sönmez G.A., Yılmaz İ. (2009). The Relationship between Isokinetic Strength of Knee Extensors/Flexors, Jumping and Anaerobic Performance. Isokinet. Exerc. Sci..

[33] Warner B., Kim K.M., Hart J.M., Saliba S. (2013). Lack of Effect of Superficial Heat to the Knee on Quadriceps Function in Individuals With Quadriceps Inhibition. J. Sport Rehabil..

[34] Marginson V., Eston R. (2001). The Relationship between Torque and Joint Angle during Knee Extension in Boys and Men. J. Sports Sci..

[35] Pincivero D.M., Salfetnikov Y., Campy R.M., Coelho A.J. (2004). Angle- and Gender-Specific Quadriceps Femoris Muscle Recruitment and Knee Extensor Torque. J. Biomech..

[36] Roberts T.J. (2016). Contribution of Elastic Tissues to the Mechanics and Energetics of Muscle Function during Movement. J. Exp. Biol..

[37] Rice D.A., McNair P.J. (2010). Quadriceps Arthrogenic Muscle Inhibition: Neural Mechanisms and Treatment Perspectives. Semin. Arthritis Rheum..

[38] Furmanek M.P., Slomka K.J., Sobiesiak A., Rzepko M., Juras G. (2018). The Effects of Cryotherapy on Knee Joint Position Sense and Force Production Sense in Healthy Individuals. J. Hum. Kinet..

[39] Kimura I.F., Thompson G.T., Gulick D.T. (1997). The Effect of Cryotherapy on Eccentric Plantar Flexion Peak Torque and Endurance. J. Athl. Train..

[40] Kim H., Lee D., Choi H.M., Park J. (2016). Joint Cooling does not Hinder Athletic Performance during High-intensity Intermittent Exercise. Int. J. Sports Med..

[41] Oosterveld F.G., Rasker J.J., Jacobs J.W., Overmars H.J. (1992). The Effect of Local Heat and Cold Therapy on the Intraarticular and Skin Surface Semperature of the Knee. Arthritis Rheum..

[42] Hohenauer E., Stoop R., Clarys P., Clijsen R., Deliens T., Taeymans J. (2018). The Effect of Pre-Exercise Cooling on Performance Characteristics: A Systematic Review and Meta-Analysis. Int. J. Clin. Med..

[43] Banfi G., Melegati G., Barassi A., Dogliotti G., d’Eril G.M., Dugue B., Corsi M.M. (2009). Effects of Whole-Body Cryotherapy on Serum Mediators of Inflammation and Serum Muscle Enzymes in Athletes. J. Biol..

[44] Partridge E.M., Cooke J., McKune A., Pyne D.B. (2019). Whole-Body Cryotherapy: Potential to Enhance Athlete Preparation for Competition?. Front. Physiol..

